# Neural correlates of episodic memory in adults with Down syndrome and Alzheimer’s disease

**DOI:** 10.1186/s13195-022-01064-x

**Published:** 2022-09-03

**Authors:** Bessy Benejam, Mateus Rozalem Aranha, Laura Videla, Concepción Padilla, Silvia Valldeneu, Susana Fernández, Miren Altuna, Maria Carmona-Iragui, Isabel Barroeta, Maria Florencia Iulita, Víctor Montal, Jordi Pegueroles, Alexandre Bejanin, Sandra Giménez, Sofía González-Ortiz, Sebastián Videla, David Bartrés-Faz, Daniel Alcolea, Rafael Blesa, Alberto Lleó, Juan Fortea

**Affiliations:** 1 Barcelona Down Medical Center, Fundació Catalana Síndrome de Down, Barcelona, Spain; 2 Memory Unit, Department of Neurology, Hospital de la Santa Creu i Sant Pau - Biomedical Research Institute Sant Pau-Universitat Autònoma de Barcelona, Barcelona, Spain; 3 Center of Biomedical Investigation Network for Neurodegenerative Diseases, Madrid, Spain; 4 Multidisciplinary Sleep Unit, Respiratory Department, Hospital de la Santa Creu i Sant Pau, Barcelona, Spain; 5 Parc de Salut, Hospital del Mar, Barcelona, Spain; 6 Clinical Research Support Unit (HUB-IDIBELL), Clinical Pharmacology Department, Bellvitge University Hospital, l’Hospitalet de Llobregat, Barcelona, Spain; 7 Pharmacology Unit, Department of Pathology and Experimental Therapeutics, School of Medicine and Health Sciences, IDIBELL, University of Barcelona, l’Hospitalet de Llobregat, Barcelona, Spain; 8 Department of Medicine, Faculty of Medicine and Health Sciences, Institute of neurosciences, University of Barcelona, and Institut d’Investigacions Biomèdiques August Pi i Sunyer (IDIBAPS), Barcelona, Spain

**Keywords:** Down syndrome, Alzheimer’s disease, Dementia, Episodic memory, MRI, Cortical thickness

## Abstract

**Background:**

Adults with Down syndrome are at an ultra-high risk of developing early-onset Alzheimer’s disease. Episodic memory deficits are one of the earliest signs of the disease, but their association with regional brain atrophy in the population with Down syndrome has not been explored. We aimed to investigate the neuroanatomical correlates of episodic memory in adults with Down syndrome and symptomatic Alzheimer’s disease.

**Methods:**

Single-center, cross-sectional study. A total of 139 adults with Down syndrome (85 asymptomatic and 54 with symptomatic Alzheimer’s disease) were included in the study (mean age 43.6 ± 10.9 years, 46% female). Episodic memory was assessed using the modified Cued Recall Test. Immediate (trial 1 free immediate recall, trial 3 free immediate recall, total free immediate recall score, and total immediate score) and delayed scores (free delayed recall score and total delayed score) were examined. Cortical thickness from magnetic resonance imaging was determined with surface-based morphometry using the FreeSurfer 6.0 software package. The clusters of reduced cortical thickness were compared between symptomatic and asymptomatic participants to create a cortical atrophy map. Then, the correlation between cortical thickness and the modified Cued Recall Test subscores were separately assessed in symptomatic and asymptomatic subjects, controlling for age, sex, and severity of intellectual disability.

**Results:**

Compared with asymptomatic participants, those with symptomatic Alzheimer’s disease showed a pattern of cortical atrophy in posterior parieto-temporo-occipital cortices. In symptomatic subjects, trial 1 immediate free recall significantly correlated with cortical atrophy in lateral prefrontal regions. Trial 3 free immediate recall and total free immediate recall were associated with the most widespread cortical atrophy. Total immediate score was related to posterior cortical atrophy, including lateral parietal and temporal cortex, posterior cingulate cortex, precuneus, and medial temporal lobe areas. Delayed memory scores were associated with cortical atrophy in temporoparietal and medial temporal lobe regions. No significant relationships were observed between episodic memory measures and cortical atrophy in asymptomatic subjects.

**Conclusions:**

Different episodic memory measures were associated with cortical atrophy in specific brain regions in adults with Down syndrome and Alzheimer’s disease. These results overlap with those described in sporadic Alzheimer’s disease and further support the similarities between Down syndrome-associated Alzheimer’s disease and that in the general population.

**Supplementary Information:**

The online version contains supplementary material available at 10.1186/s13195-022-01064-x.

## Background

Down syndrome (DS) is the most frequent genetic form of intellectual disability [1] and is associated to an increased risk of developing early Alzheimer’s disease (AD) dementia due to the presence of an extra copy of the amyloid β precursor protein gene, which is coded on chromosome 21, and is thus overexpressed in people with DS [2].

Episodic memory refers to the ability to learn, store, and retrieve information about unique personal experiences that occur in daily life [3]. Episodic memory impairment is the hallmark symptom of typical Alzheimer’s disease (AD) in the general population and has been associated with early atrophy of medial temporal lobe structures [4]. As the disease progresses, AD pathology spreads to other cortical regions, including ventral and lateral temporal cortex, followed by parietal and frontal cortices [4, 5], affecting other cognitive domains. In DS, neurodegenerative changes due to AD follow a similar pattern as those described in sporadic AD, although it occurs at a younger age [6–8]. Early longitudinal studies suggested executive dysfunction and behavioral and psychological symptoms of dementia as commonly observed symptoms during the early stages of AD in DS [9], although the identification of imaging and fluid biomarkers in the past 5 years has provided new opportunities to characterize the sequence of cognitive decline during the preclinical and prodromal stages of the disease in this population [10]. Recent research now indicates that individuals with DS have a similar clinical presentation to that of people with sporadic or autosomal dominant AD, more specifically, declines in episodic memory and attention measures early in the course of the disease [11, 12].

Although medial temporal lobe regions play a central role in episodic memory, other cortical regions are also necessary to accurately remember new information. More specifically, three different processes or stages have been described in episodic memory that are sustained by different brain networks: encoding, retrieval, and storage [13]. In the general population, these memory processes have been assessed with the Free and Cued Selective Reminding Test (FCSRT) through different subscores and have been associated with different patterns of brain atrophy in patients with mild AD. Specifically, encoding has been associated with atrophy in parietal and temporal regions, storage with atrophy in entorhinal and parahippocampal regions, and retrieval with atrophy in a widespread network encompassing frontal regions [13]. Similarly, Wolk et al. investigated the relationship between performance on different stages of the Rey Auditory Verbal Learning Test (RAVLT), a verbal episodic memory test, and structural brain measures in patients with mild AD from the general population, including immediate and delayed memory recall [14]. This study also found dissociable relationships between regional brain atrophy and different aspects of memory function, indicating that different cognitive processes support different stages of episodic memory performance.

In DS, episodic memory has been evaluated using adapted tests such as the modified Cued Recall Test (mCRT), which has demonstrated to be useful to detect memory decline due to early-stage AD [15] and, more recently, for the diagnosis of prodromal AD [16]. The mCRT has emerged as a promising cognitive indicator of transition to the preclinical and prodromal stage of AD in subjects with DS [11]. Furthermore, poor performance on this episodic memory test has recently been related to white matter degeneration prior to the onset of dementia in adults with DS [17]. The association between regional cortical atrophy and different stages of episodic memory performance in patients with DS and symptomatic AD has not yet been studied.

The aim of this study was to investigate the neural correlates of different episodic memory processes measured with the mCRT in a large cohort of adults with DS, with and without symptomatic AD. Understanding the anatomic basis of the processes underlying episodic memory has potential practical implications, as it may facilitate the integration of clinical and radiological information, helping increase the diagnostic accuracy of AD in this population.

## Methods

### Study design and participants

In this single-center cross-sectional study, adults with DS aged 18 years or older were recruited from a population-based health plan designed to screen for AD dementia. This health plan includes an annual neurological and neuropsychological assessment, undertaken at the Alzheimer-Down Unit from the Catalan Down Syndrome Foundation and Hospital of Sant Pau, in Barcelona, Spain. Individuals who are also interested in participating in research studies are included in the Down Alzheimer Barcelona Neuroimaging Initiative (DABNI) cohort. The aim of the DABNI project is to improve our understanding of the natural history and mechanisms that drive AD in DS. It includes multimodal biomarker assessments, comprising magnetic resonance imaging (MRI), positron emission tomography, lumbar puncture, sleep studies, and blood and genetic analyses.

We included adults with DS (mild or moderate intellectual disability) with available MRI and a complete neuropsychological assessment. Patients showing any psychiatric or medical disorder that could affect cognition and/or functionality were excluded. We also excluded those patients not able to complete the mCRT due to severe cognitive decline or due to severe or profound intellectual disability [16].

The study was approved by the Sant Pau Ethics Committee following the standards for medical research in humans recommended by the Declaration of Helsinki and reported to the Ministry of Justice, according to the Spanish law for research in people with intellectual disabilities. All participants or their legally authorized representative gave written informed consent before enrollment.

### Neuropsychological assessment

Caregivers completed a semi-structured adapted health questionnaire, the Cambridge Examination for Mental Disorders of Older People with Down syndrome and others with intellectual disabilities (CAMDEX-DS), aimed to evaluate cognitive decline in people with intellectual disability [18].

The neuropsychological test battery included the Cambridge Cognitive Examination for Older Adults with Down’s syndrome (CAMCOG-DS), a neuropsychological test battery included in the CAMDEX-DS, comprising subscales for the assessment of the following cognitive domains: orientation, language, memory, attention, praxis, abstract thinking, and perception. For the assessment of episodic memory, the mCRT was used (see below).

### Level of intellectual disability

The level of intellectual disability was categorized according to the Diagnostic and Statistical Manual of Mental Disorders, Fifth Edition [19] as mild, moderate, severe, or profound intellectual disability, based on caregiver’s reports of the individuals’ best-ever level of functioning.

### Assessment of episodic memory

The mCRT is an adapted test to assess episodic memory in people with intellectual disability [15]. This test was selected because it is based on semantic cueing, which allows controlled encoding processes and facilitates retrieval from stored information. The mCRT was administered according to the procedure described by Devenny et al. [15]. Briefly, participants are asked to memorize 12 stimuli (black-and-white line drawings of objects representing a distinct semantic category) presented on three, 4-item cards. The test consists of three trials of free and cued recall performed immediately after the learning phase to compute a total free immediate recall score (FIRS) and a total immediate score (FIRS + cued recall; TIS). The maximum score for both measures is 36. The same recall procedure (free and cued) is done after a 20-min delay to compute a free delayed recall score (FDRS) and a total delayed score (FDRS + cued recall; TDS). The maximum score for both delayed scores is 12. In the general population, some authors found that early immediate learning trials rely on different brain regions than later immediate learning trials [14]; therefore, we also analyzed the data for trial 1 free immediate recall (T1) and trial 3 free immediate recall (T3), representing early and late immediate learning, respectively.

### Diagnostic categories

As in previous studies [6, 20], participants with DS were classified by the neurologists and neuropsychologists who assessed them in a consensus meeting into the following groups: asymptomatic, in those with no clinical or neuropsychological suspicion of AD; prodromal AD, in those who had cognitive decline, but symptoms did not fulfill criteria for dementia; or AD dementia, in those who had cognitive decline that significantly interfered in subject’s activities of daily living. In this study, participants were divided into symptomatic (including prodromal AD and AD dementia) and asymptomatic groups.

### Structural MRI

All participants underwent MRI in a 3-T Philips Achieva (Philips Healthcare) or a 3-T Siemens Prisma (Siemens Healthcare) MR scanner (protocols detailed in the supplements). Cortical thickness was assessed with surface-based analysis in FreeSurfer 6.0 (https://surfer.nmr.mgh.harvard.edu/) using an automated standard pipeline detailed elsewhere [21–23]. Briefly, this pipeline normalizes T1-weighted images to a standard space (MNI305), removes non-brain tissue, classifies each brain voxel as gray of white matter, and defines interfaces between gray and white matter (GM and WM, respectively) and between GM and cerebrospinal fluid (CSF), generating WM and pial surfaces, respectively. To correct for eventual defects on these surfaces, manual edits were performed when needed. The cortical thickness is given by the distance between WM and pial surfaces at each point. Finally, each surface was smoothed with a Gaussian filter of 15mm before statistical analysis.

### Statistical analysis

#### Demographic data

Differences between asymptomatic and symptomatic subjects in demographic characteristics and between the episodic memory scores were assessed using Student’s *t*-test. The chi-squared test was used for testing relationships on categorical variables (sex and level of intellectual disability). A *p-*value of ≤ 0.05 was considered significant. The statistical analyses were performed using IBM SPSS Statistics Software (version 22).

#### Neuroimaging data

Initially, cortical thickness was compared between asymptomatic and symptomatic individuals with DS using FreeSurfer’s QDEC (Query, Design, Estimate, Contrast) interface in order to identify regions where cortical thickness is significantly reduced in symptomatic subjects and create a cortical atrophy map. Sex, age, and degree of intellectual disability were included as covariates. We then performed correlation analyses between the different memory performance scores and cortical thickness using a whole-brain vertex-wise analysis separately in asymptomatic and symptomatic participants with QDEC. To this purpose, a general linear model was applied, controlling for sex, age, and degree of intellectual disability. The results were corrected for multiple comparisons using Monte Carlo null-Z simulation (cluster analysis) as implemented in QDEC (*p corrected* = 0.05) [24].

## Results

### Demographic characteristics

Between May 1, 2013, and Jun 7, 2021, 148 participants met the inclusion criteria and were included in this study (see study flowchart in Supplementary Fig. 1). From this group, 9 participants with scan artifacts impairing automated image processing had to be excluded. From the remaining sample (139 participants), 85 were asymptomatic and 54 met criteria for prodromal AD or AD dementia (28 and 26 subjects, respectively. These subjects constituted the symptomatic AD group).

Demographic characteristics and episodic memory scores for these groups are shown in Table 1. As expected, symptomatic participants were significantly older and had lower performance on all mCRT scores than the asymptomatic participants (*p* < 0.001). There were no differences in sex (*p =* 0.46) or severity of intellectual disability (*p* = 0.29) between the asymptomatic and symptomatic groups.

**Table 1:** Demographic characteristics and episodic memory scores

	All participants	Asymptomatic	Symptomatic	*p-*values
	(*N* = 139)	(*N* = 85)	(*N* = 54)	
Age (mean, sd)	43.6 (10.9)	38.5 (10.1)	52.2 (4.6)	<0.001
Female sex, *n* (%)	64 (46)	37 (43.5)	27 (50)	0.456
Severity of ID, *n* (%)				0.288
Mild	46 (33.1)	31 (36.5)	15 (27.8)	
Moderate	93 (66.9)	54 (63.5)	39 (72.2)	
Time between MRI and mCRT, days (mean, sd)	108.2 (81.4)	116.2 (79.0)	95.5 (84.2)	0.088
mCRT scores (mean, sd)				
Trial 1 free recall (0–12)	4.3 (2.3)	5.4 (1.9)	2.5 (1.7)	<0.001
Trial 3 free recall (0–12)	5.8 (2.9)	7.7 (1.7)	2.7 (2.6)	<0.001
Free immediate recall score (0–36)	15.3 (7.4)	19.8 (4.7)	7.7 (5.3)	<0.001
Total immediate score (0–36)	29.5 (9.3)	35 (1.6)	20.7 (9.9)	<0.001
Free delayed recall score (0–12)	5 (3.5)	6.8 (2.7)	1.7 (2.4)	<0.001
Total delayed score (0–12)	9.7 (3.3)	11.5 (1.0)	6.3 (3.6)	<0.001

*p* values are given for comparison between asymptomatic and symptomatic subjects. Age refers to the age recorded on the day of the mCRT administration. Brackets next to each episodic memory measure reflect the total possible range of scores

*ID* Intellectual disability, *mCRT* Modified Cued Recall Test

### Pattern of cortical atrophy in participants with DS and symptomatic AD

**Fig. 1:**
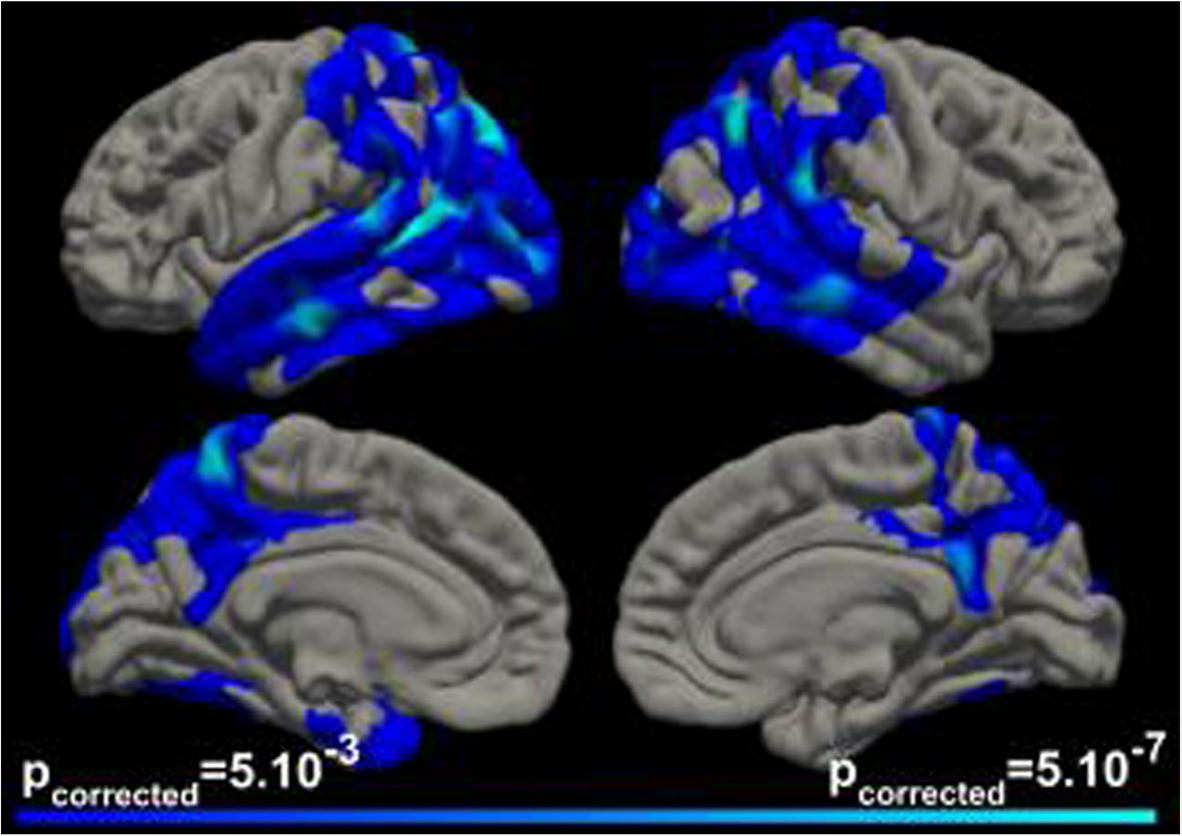
Pattern of cortical atrophy in symptomatic DS compared to asymptomatic DS participants. The clusters in blue-cyan scale represent the brain regions of statistically significant cortical atrophy in subjects with symptomatic Alzheimer’s disease (AD) compared to asymptomatic participants, after correction for multiple comparisons (threshold for significance *p* corrected = 0.05)

Compared with asymptomatic adults with DS, individuals with DS and symptomatic AD showed cortical atrophy in the temporoparietal, precuneus-posterior cingulate, and occipital areas (Fig. 1).

### Cortical thickness and memory performance

#### Immediate recall

**Fig. 2:**
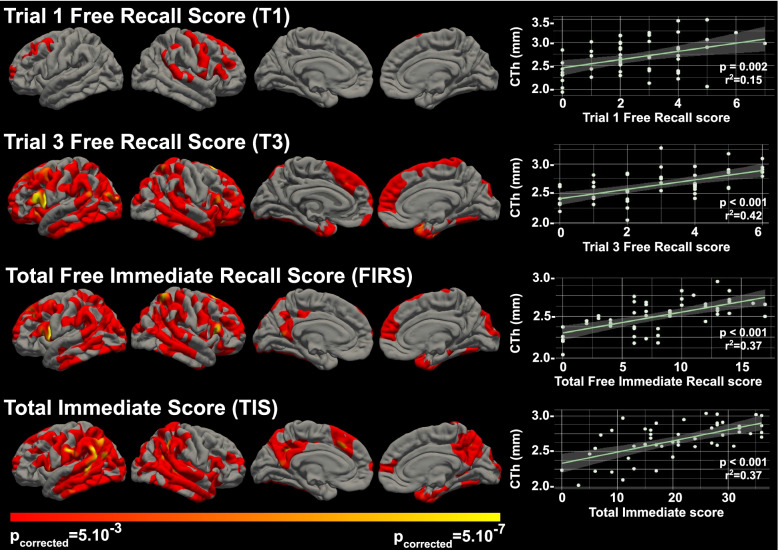
Correlation analyses between cortical thickness and immediate memory scores in symptomatic participants. The clusters in red-yellow scale represent the brain regions where each score was significantly related to cortical thickness after correction for multiple comparisons (threshold for significance *p* corrected = 0.05). Scatter plots on the right show the correlation between each score and the cortical thickness at the most significant vertex. CTh, cortical thickness (in millimeters)

Figure 2 shows the correlation analyses between cortical thickness and the immediate memory scores (T1, T3, FIRS, and TIS) in symptomatic participants. T1 scores showed a significantly positive relationship with cortical thickness in the lateral prefrontal regions of both hemispheres. Performance on T3 correlated positively with cortical thickness in a more widespread network encompassing lateral regions in frontal, temporal, parietal, and occipital lobes, bilaterally, with the most significant clusters located in the left middle frontal, inferior frontal, supramarginal, and lingual gyri, and in the right temporal pole and superior frontal gyrus. T3 in addition also correlated with a small cluster located in the right parahippocampal cortex.

The distribution of the clusters with significant association between cortical thickness and FIRS resembled that observed for T3, but with more widespread cortical atrophy also involving the right inferior frontal gyrus and superior parietal lobule, as well as the left posterior cingulate gyrus and precuneus.

TIS was mostly associated with cortical thickness in bilateral parieto-temporal regions, involving both precuneus and posterior cingulate gyri, and the right medial temporal lobe structures, namely the entorhinal and parahippocampal cortex (with *p* ≤ 0.05) (Fig. 2).

In asymptomatic participants, no clusters of significant association between immediate memory scores and cortical thickness were observed (Supplementary Fig. 2).

#### Delayed recall

**Fig. 3:**
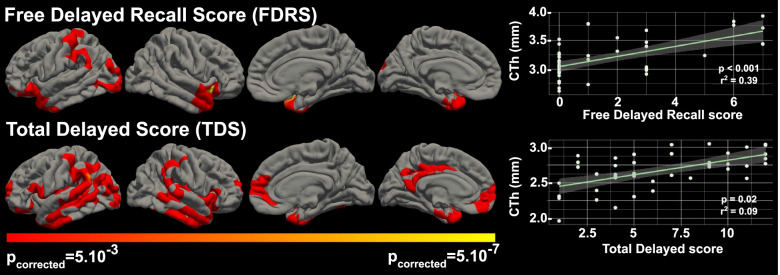
Correlation analyses between cortical thickness and delayed memory scores in symptomatic participants. The clusters in red-yellow scale represent the brain regions where each score was significantly related to cortical thickness after correction for multiple comparisons (threshold for significance *p* corrected = 0.05). Scatter plots on the right show the correlation between each score and the cortical thickness at the most significant vertex. CTh, cortical thickness (in millimeters)

Figure 3 shows the correlation between cortical thickness and delayed memory scores (FDRS and TDS) in symptomatic participants. FDRS was associated with cortical thickness in areas mainly localized in anterior lateral and medial temporal regions, including both temporal poles, left entorhinal cortex, and right lateral superior and middle temporal gyri. We also found significant associations with cortical thickness in both inferior frontal gyri and in left postcentral and supramarginal gyri (*p* ≤ 0.05).

TDS was significantly associated with cortical thickness in medial and lateral temporal lobe structures of both hemispheres. Clusters of significant correlation were also found in both supramarginal gyri, in the medial face of both superior frontal gyri, and in the left posterior cingulate gyrus (*p*≤0.05).

We did not find clusters of significant correlations between cortical thickness and delayed memory measures in asymptomatic participants (Supplementary Fig. 2).

## Discussion

To our knowledge, this is the first study that investigates the neuroanatomical correlates of different processes involved in episodic memory in a cohort of adults with DS. Using structural MRI and an adapted episodic memory test, the mCRT, we found different correlations between the subtests assessing the different episodic memory processes and cortical thickness in symptomatic AD, suggesting that different anatomical regions play unique roles at different stages of episodic memory processing.

Adults with DS and symptomatic AD showed a pattern of cortical atrophy in lateral parieto-temporo-occipital cortices and in the posterior cingulate and precuneus. This pattern of widespread posterior cortical thinning has been previously described in amyloid-positive individuals with DS [25] and is very similar to that observed in sporadic AD [26].

Relationships in symptomatic individuals are detailed hereafter. The correlations between episodic memory measures and cortical thickness extended well beyond the medial temporal lobe structures. The different mCRT subscores were associated with cortical atrophy in different regions. T1 immediate free recall, a score that reflects initial encoding processes [13], mainly involved frontal lobe areas. FIRS, a score used to assess immediate retrieval [13], was associated with a widespread cortical network encompassing lateral frontal, parietal, temporal, and occipital regions, bilaterally, while TIS, which reflects immediate storage [13], was associated with more posterior cortical atrophy, including regions in the lateral parietal, temporal and occipital cortices, precuneus, posterior cingulate cortex, and medial temporal lobe cortices. In contrast to the immediate memory trials, delayed memory performance was related to a less distributed pattern of cortical atrophy, predominantly involving lateral and medial temporal lobe regions. Importantly, these associations were only seen in symptomatic participants and not in asymptomatic, possibly reflecting the smaller dynamic range of the mCRT scores and the milder atrophy in asymptomatic individuals [16]. To our knowledge, no other studies have related cortical thickness to different episodic memory measures in asymptomatic individuals with DS. In the general population, some authors have attempted to correlate FCSRT performance with volumetric measures of the hippocampal formation or entorhinal cortex in healthy aging subjects, without finding significant associations [27, 28].

Episodic memory impairment is a hallmark symptom of sporadic AD and DS-related AD during the early and middle stages of the disease [29–31] and has been traditionally associated with medial temporal lobe damage [32–34]. In the last decades, a growing number of neuroimaging studies have demonstrated that episodic memory does not depend solely on medial temporal structures. Several cognitive processes, like language, working memory, semantic processing, attention, and executive functions, which rely upon distinct brain networks, are necessary for successful episodic memory [13, 14, 35, 36]. We found that performance on T1 of the mCRT was associated with cortical thickness in frontal lobe regions, but not in medial temporal lobe structures in subjects with symptomatic AD, thus indicating that initial encoding was more dependent on regions supporting cognitive functions such as attention, working memory, or executive functions. Ventrolateral and dorsolateral prefrontal cortex have been described to implement control processes that support memory encoding (e.g., selection processes that direct attention toward goal-relevant information or organizational processes necessary for optimal memory encoding, respectively) [37, 38]. Our results are in line with those reported by Wolk et al. in sporadic AD, who also found a lack of influence of medial temporal structures during early list learning trials in patients with mild AD from the general population [14].

The temporal pole consistently appeared to be related to immediate and delayed memory measures (with the exception of T1 immediate recall) in our study. In this sense, functional neuroimaging studies have related the anterior temporal lobes to semantic processing (mental representations of the meaning of words, objects, people, and factual information about the world) [39, 40]. The semantic organization of the information that has to be encoded allows for a deeper processing and thus facilitates posterior retrieval processes. Our results are again consistent with previous studies relating the atrophy of the temporal pole with poor immediate recall performance in patients with early-stage AD in the general population [14, 35].

In addition, episodic memory performance (particularly T3, FIRS and TIS immediate recall scores, and TDS) was related to cortical thickness in the lateral posterior parietal cortex (PPC). These areas have been implicated in episodic memory retrieval because of their role in attentional processes (attention to internal representations). The dorsal PPC is implicated in the voluntary orienting of attention to relevant stimuli based on retrieval goals (top-down attentional processes), while the ventral PPC responds to the detection of new behaviorally relevant stimuli which are outside the focus of attention defined by the dorsal PPC (bottom-up attentional processes) [41, 42]. Moreover, inferior parietal regions (together with regions within the inferior frontal gyrus) have been associated with the storage function of the phonological loop and the visuospatial sketchpad supporting speech-based and visuospatial working memory, respectively [43]. Working memory, which temporarily maintains and stores information, is crucial for the transfer of information into a long-term store. Other regions also reported to be supportive of working memory are the posterior superior temporal gyrus, the premotor cortex, and the dorsolateral prefrontal cortex [44], regions found to be strongly associated with immediate memory measures in our study.

Atrophy in medial temporal lobe (MTL) structures, in particular the entorhinal and parahippocampal cortices, was found to be related to TIS and TDS. Low scores on both measures reflect storage deficits (immediate and delayed storage, respectively). Many studies support the role of MTL regions in the acquisition and maintenance of episodic memory [3, 13, 36]. The association between TIS and atrophy of extrahippocampal MTL regions in the right hemisphere in our study is not in line with previous research using the FCSRT or the RAVLT in patients with sporadic AD [35, 36]. One possible explanation for these discrepancies could be the visual nature of the stimuli used in the present study, unlike the FCSRT used in the general population, which is based on the learning of verbal stimuli. Similarly, we found that immediate and delayed recall measures were also associated with atrophy in occipital regions. Ventral occipito-temporal regions have been related to visual object processing [45]. Poor performance on some mCRT measures, which is based on the recall of familiar line drawings, could partly be explained by damage to this visual processing pathway.

Performance on the TIS, and to a lesser extent the TDS, was also associated with posteromedial regions, namely the precuneus and the posterior cingulate cortex. Recent functional imaging studies have suggested that memory function is subserved by a set of distributed networks which include the medial temporal lobe system and a set of cortical regions collectively referred to as the default mode network (DMN). The regions of the DMN include the anteromedial prefrontal cortex, the posterior cingulate cortex, the precuneus, the angular gyrus, and the medial temporal lobe [46]. Some authors have demonstrated that early amyloid deposition in sporadic AD occurs in areas mainly located within the DMN [47] and that this network plays a key role orienting the focus of attention to stored representational knowledge [48]. In DS subjects, recent research found that reductions in DMN connectivity to posterior brain regions were linked to the presence of AD neuropathology in a pattern substantially similar to that seen in sporadic AD [49, 50], even in asymptomatic individuals. Nevertheless, the association between the effects of AD neuropathology on the DMN and performance on episodic memory measures in the DS population has not been described.

Our results have several implications. First, the pattern of cortical atrophy observed in individuals with DS and AD in our study is remarkably similar to that described in the general population with AD [26], indicating that AD in individuals with DS targets the same cortical regions affected in sporadic AD. Second, the cortical areas found to be related to episodic memory in our study are in line with those previously described in sporadic AD. Our results thus reinforce the similarities between AD in DS and sporadic AD. Third, our results also support the use of the mCRT as a potential neuropsychological marker for the diagnosis of AD in people with DS, and particularly the TIS and the TDS (measures reflecting storage deficits), as these scores correlated well with medial temporal lobe regions, areas considered to be the earliest affected by AD neuropathology.

Our study has several strengths, including the large sample size of subjects included in our study with symptomatic AD, as well as the fact that our participants come from a large population-based cohort of adults with DS.

## Limitations

We also acknowledge some limitations. First, this is a single-center study and did not include an age-matched control group of euploid individuals to test the neurodevelopmental abnormalities. Thus, our results need to be replicated in other cohorts to confirm the generalizability of our results. Second, as patients with prodromal AD and AD dementia were analyzed together, it has not been possible to characterize AD in its earliest stages. Future studies should include a more homogeneous sample of subjects with prodromal AD to better characterize the early stages of AD in this population.

## Conclusions

Our results provide new evidence about the neuroanatomical correlates of the episodic memory deficits in adults with DS and symptomatic AD that largely corresponds with the findings described in sporadic AD. This study further supports the similarities of the AD pathophysiological process in sporadic AD and in DS.

## Supplementary Information

**Additional file 1: Supplementary Figure 1.** Study flow chart. Description of data: flow chart of included and excluded subjects. Footnote: ID, intellectual disability; mCRT, modified Cued Recall Test; MRI, magnetic resonance imaging; OCD, obsessive compulsive disorder.

**Additional file 2.** MRI acquisition protocols.

**Additional file 3: Supplementary Figure 2.** Correlation analyses between cortical thickness and immediate and delayed memory scores in asymptomatic participants. Legend: Brain regions where each score was significantly related to cortical thickness (threshold for significance p corrected = 0.05, after correction for multiple comparisons). No clusters of significant correlation between cortical thickness and immediate or delayed memory scores were observed in asymptomatic participants.

## Data Availability

The anonymized data used and/or analyzed during the current study are available from the corresponding author upon reasonable request.

## Abbreviations

AD: Alzheimer’s disease
CAMCOG-DS: Cambridge Cognitive Examination for Older Adults with Down’s syndrome
CAMDEX-DS: Cambridge Examination for Mental Disorders of Older People with Down syndrome and others with intellectual disabilities
CSF: Cerebrospinal fluid
DABNI: Down Alzheimer Barcelona Neuroimaging Initiative
DMN: Default mode network
DS: Down’s syndrome
FCSRT: Free and Cued Selective Reminding Test
FDRS: Free delayed recall score
FIRS: Free immediate recall score
GM: Gray matter
ID: Intellectual disability
mCRT: Modified Cued Recall Test
MRI: Magnetic resonance imaging
MTL: Medial temporal lobe
PPC: Posterior parietal cortex
QDEC: Query, Design, Estimate, Contrast
RAVLT: Rey Auditory Verbal Learning Test
T1: Trial 1 free immediate recall
T3: Trial 3 free immediate recall
TDS: Total delayed score
TIS: Total immediate score
WM: White matter
