# Supplementary material for: Neural correlates of episodic memory in adults with Down syndrome and Alzheimer’s disease
Source: Alzheimers Res Ther. 2022 Sep 3;14:123. doi: 10.1186/s13195-022-01064-x (PMC9440567; doi:10.1186/s13195-022-01064-x)
Supplement: Supplementary file 1 — Additional file 1: Supplementary Figure 1. Study flow chart. Description of data: flow chart of included and excluded subjects. Footnote: ID, intellectual disability; mCRT, modified Cued Recall Test; MRI, magnetic resonance imaging; OCD, obsessive compulsive disorder. [file 13195_2022_1064_MOESM1_ESM.pptx]

## Slide 1
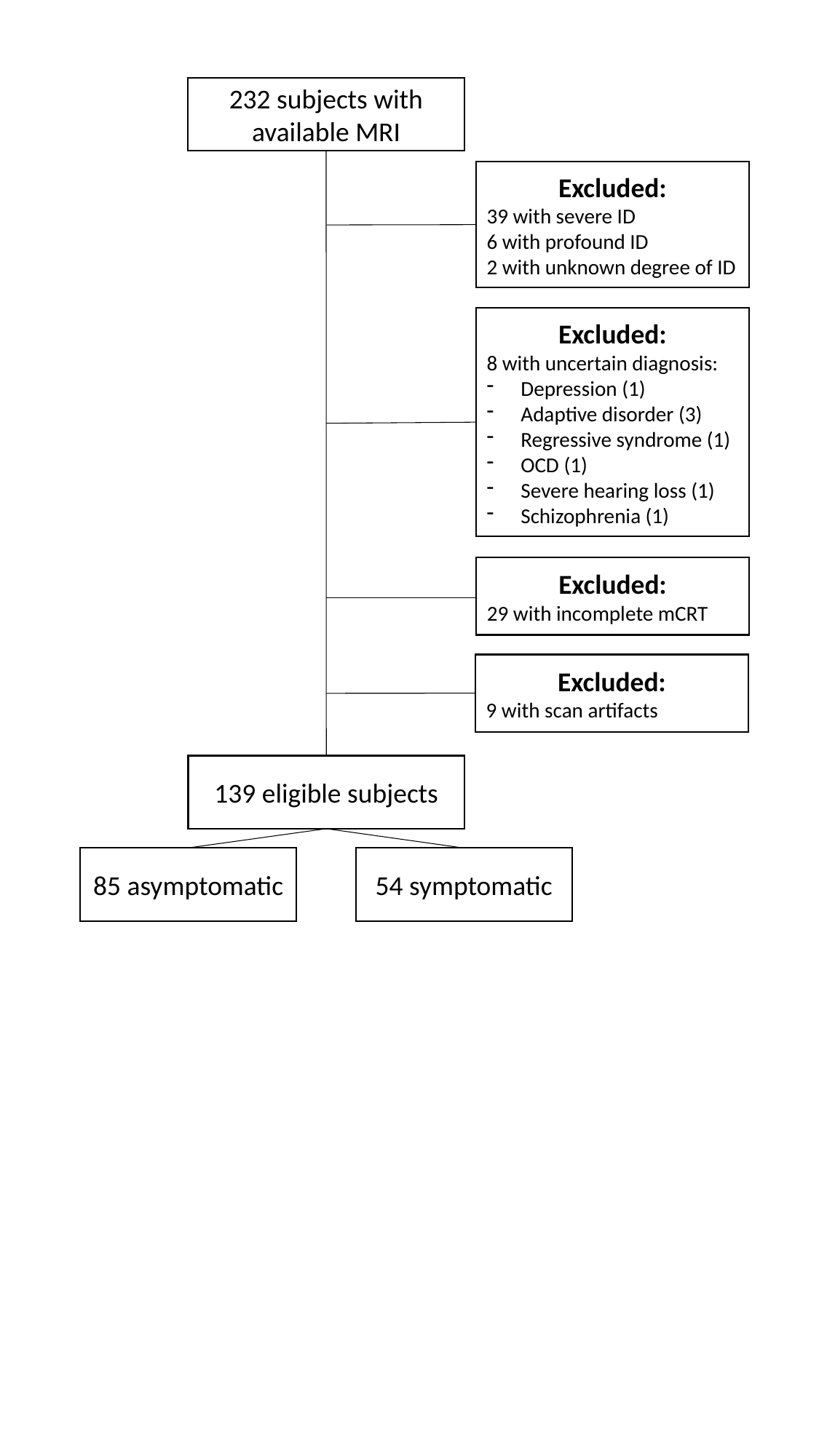

232 subjects with available MRI
Excluded:
39 with severe ID
6 with profound ID
2 with unknown degree of ID
Excluded:
8 with uncertain diagnosis:
Depression (1)
Adaptive disorder (3)
Regressive syndrome (1)
OCD (1)
Severe hearing loss (1)
Schizophrenia (1)
Excluded:
29 with incomplete mCRT
Excluded:
9 with scan artifacts
139 eligible subjects
85 asymptomatic
54 symptomatic
