# Supplementary figures and images for: Neural correlates of episodic memory in adults with Down syndrome and Alzheimer’s disease
Source: Alzheimers Res Ther. 2022 Sep 3;14:123. doi: 10.1186/s13195-022-01064-x (PMC9440567; doi:10.1186/s13195-022-01064-x)

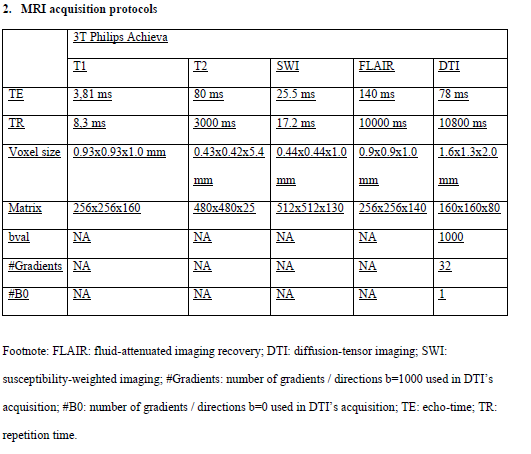


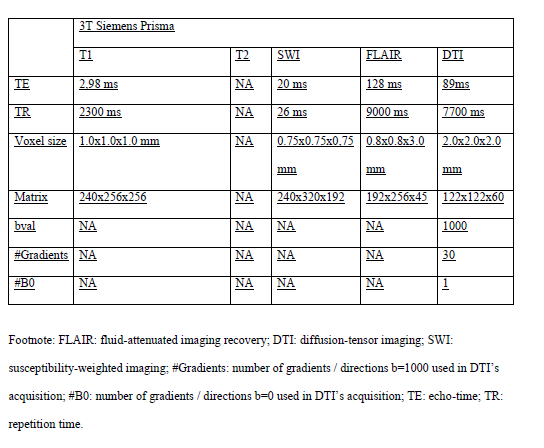

Supplement: Supplementary file 2 — Additional file 2. MRI acquisition protocols. [file 13195_2022_1064_MOESM2_ESM.docx]

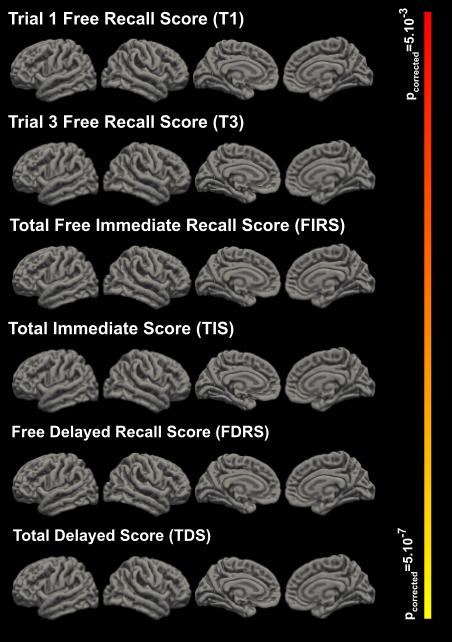

Supplement: Supplementary file 3 — Additional file 3: Supplementary Figure 2. Correlation analyses between cortical thickness and immediate and delayed memory scores in asymptomatic participants. Legend: Brain regions where each score was significantly related to cortical thickness (threshold for significance p corrected = 0.05, after correction for multiple comparisons). No clusters of significant correlation between cortical thickness and immediate or delayed memory scores were observed in asymptomatic participants. [file 13195_2022_1064_MOESM3_ESM.jpg]
